# Influenza Virus Down-Modulates G6PD Expression and Activity to Induce Oxidative Stress and Promote Its Replication

**DOI:** 10.3389/fcimb.2021.804976

**Published:** 2022-01-06

**Authors:** Marta De Angelis, Donatella Amatore, Paola Checconi, Alessandra Zevini, Alessandra Fraternale, Mauro Magnani, John Hiscott, Giovanna De Chiara, Anna Teresa Palamara, Lucia Nencioni

**Affiliations:** ^1^ Laboratory Affiliated to Istituto Pasteur Italia-Fondazione Cenci Bolognetti, Department of Public Health and Infectious Diseases, Sapienza University of Rome, Rome, Italy; ^2^ Scientific Department, Army Medical Center, Via di Santo Stefano Rotondo, Rome, Italy; ^3^ Department of Human Sciences and Promotion of the Quality of Life, San Raffaele Roma Open University, IRCCS San Raffaele Roma, Rome, Italy; ^4^ Pasteur Laboratory, Istituto Pasteur Italia—Fondazione Cenci Bolognetti, Rome, Italy; ^5^ Department of Biomolecular Sciences, University of Urbino Carlo Bo, Urbino, Italy; ^6^ Institute of Translational Pharmacology, National Research Council, Rome, Italy; ^7^ Department of Infectious Diseases, Istituto Superiore di Sanità, Rome, Italy

**Keywords:** influenza virus, redox state, oxidative stress, glutathione, G6PD, NRF2, SIRT2, antioxidant response

## Abstract

Influenza virus infection induces oxidative stress in host cells by decreasing the intracellular content of glutathione (GSH) and increasing reactive oxygen species (ROS) level. Glucose-6-phosphate dehydrogenase (G6PD) is responsible for the production of reducing equivalents of nicotinamide adenine dinucleotide phosphate (NADPH) that is used to regenerate the reduced form of GSH, thus restoring redox homeostasis. Cells deficient in G6PD display elevated levels of ROS and an increased susceptibility to viral infection, although the consequences of G6PD modulation during viral infection remain to be elucidated. In this study, we demonstrated that influenza virus infection decreases G6PD expression and activity, resulting in an increase in oxidative stress and virus replication. Moreover, the down regulation of G6PD correlated with a decrease in the expression of nuclear factor erythroid 2-related factor 2 (NRF2), a key transcription factor that regulates the expression of the antioxidant response gene network. Also down-regulated in influenza virus infected cells was sirtuin 2 (SIRT2), a NADPH-dependent deacetylase involved in the regulation of G6PD activity. Acetylation of G6PD increased during influenza virus infection in a manner that was strictly dependent on SIRT2 expression. Furthermore, the use of a pharmacological activator of SIRT2 rescued GSH production and NRF2 expression, leading to decreased influenza virus replication. Overall, these data identify a novel strategy used by influenza virus to induce oxidative stress and to favor its replication in host cells. These observations furthermore suggest that manipulation of metabolic and oxidative stress pathways could define new therapeutic strategies to interfere with influenza virus infection.

## Introduction

Viral infection is associated with alterations in the intracellular redox balance which are important for the activation of redox-sensitive pathways required for viral replication (Checconi et al., 2020). As an example, influenza A virus, like many viruses, causes an imbalance in the intracellular redox state, characterized by depletion of glutathione (GSH) and an increase in nicotinamide adenine dinucleotide phosphate (NADPH) oxidase 4 (NOX4)-mediated reactive oxygen species (ROS) production. The following pro-oxidant state has been shown to favor different steps of viral replication including the folding of surface glycoproteins and the nucleus-cytoplasmic traffic of viral ribonucleoprotein (Cai et al., 2003; Nencioni et al., 2003; Sgarbanti et al., 2011; To et al., 2014; Amatore et al., 2015).

Glucose-6-phosphate dehydrogenase (G6PD) is the rate-limiting enzyme of the pentose phosphate pathway; G6PD plays a central role in cellular physiology, as it is a major source of NADPH that is required by many essential cellular systems to maintain intracellular redox state equilibrium (Yang et al., 2016). Indeed, NADPH is used by the glutathione reductase enzyme to regenerate GSH, the main intracellular antioxidant (Dickinson and Forman, 2002; Forman et al., 2009; van Zwieten et al., 2014).

G6PD deficiency has been associated to an increased susceptibility to Hepatitis A and E virus infections (Miri-Aliabad et al., 2017; Ahmad et al., 2018), while other studies demonstrated that G6PD-deficient cells supported enterovirus 71 replication more efficiently than control G6PD-expressing cells (Ho et al., 2008). In G6PD-knockdown cells, higher ROS production correlated with increased human coronavirus (HCoV) 229E replication (Wu et al., 2008), and it was recently suggested that individuals affected by G6PD deficiency may be more susceptible to SARS-CoV-2 infection (Buinitskaya et al., 2020; Jain et al., 2020; Vick, 2020). Overall, these data indicate that G6PD levels play an important role in regulating the susceptibility to viral infection and the extent of viral replication. How G6PD expression and enzymatic activity may be modulated during influenza virus infection remains unexplored, as does its role in virus-induced oxidative stress and viral replication.

The expression of G6PD is regulated predominantly by the intracellular antioxidant response network, which is controlled in turn by the transcription factor nuclear factor erythroid 2-related factor 2 (NRF2) (Rojo de la Vega et al., 2018; Baird and Yamamoto, 2020). Normally, NRF2 is retained in the cytoplasm in association with Kelch like-ECH-associated protein 1 (KEAP1) that promotes NRF2 degradation *via* the Cullin 3 complex and the ubiquitin-proteasome pathway. Oxidative stress-induced ROS accumulation disrupts the NRF2-KEAP1 complex, leading to nuclear translocation of NRF2 and transcriptional induction of antioxidant response genes, including those involved in the GSH synthesis, ROS elimination and NADPH production (Shaw and Chattopadhyay, 2020). Moreover, it was reported that the modulation of pentose phosphate pathway, as well as G6PD expression, can regulate NRF2 pathway activation, implying that G6PD and NRF2 may crosstalk with each other to maintain cellular redox homeostasis (Heiss et al., 2013).

In addition to transcriptional induction of G6PD expression by NRF2, G6PD enzymatic activity is regulated by post-translational modifications including lysine acetylation (Kim et al., 2006; Choudhary et al., 2009). Sirtuin 2 (SIRT2), a member of the sirtuin NAD+-dependent deacetylase family (Singh et al., 2018), has been shown to regulate G6PD deacetylation during oxidative stress. Indeed, SIRT2 mediates deacetylation of G6PD at lysine 403 (K403) and promotes NADPH production, thus restoring antioxidant defense and increasing the levels of GSH (Wang et al., 2014; Xu et al., 2016).

In the present study, we investigated: 1) whether influenza virus modulates G6PD expression and activity; and 2) whether G6PD modulation plays a role in virus-induced redox changes and viral replication. Our data identify G6PD modulation as a novel mechanism exploited by influenza virus to induce oxidative stress in host cells and favor its replication and suggest that the rescue of antioxidant response could represent a new cell-targeted strategy to interfere with influenza virus infection.

## Materials and Methods

### Cell Cultures and Infection

A549 (Human Lung Carcinoma cells), MDCK (Madin-Darby Canine Kidney cells), HEK293 (Human Embryo Kidney cells) and BEAS-2B (Human Lung Epithelial cells) were grown in Dulbecco’s modified Eagle’s medium (DMEM) or in Minimum Essential Medium (MEM) supplemented with 10% fetal bovine serum (FBS), 0.3 mg/ml glutamine, 100 U/ml penicillin, and 100 μg/ml streptomycin. Confluent monolayer of cells was challenged with influenza A virus strain human A/Puerto Rico/8/34 H1N1 (PR8), A/California/7/2009/H1N1 (pH1N1) or avian Parrot/Ulster/73 H7N1 (H7N1) influenza virus strains at 0.3 MOI of infection for 1 h at 37°. After the viral adsorption, the cells were washed with phosphate-buffered saline (PBS) and then incubated with medium supplemented with 2% FBS for 24 h. Virus production was determined in the supernatants of infected cells, by measuring the hemagglutinating units (HAU) or the tissue culture infectious dose 50 (TCID50) as previously described (Nencioni et al., 2003).

### Silencing of G6PD in A549 Cells

A549 cells were seeded in 12-well culture plates at a density of 5x10^5^ cells per well in DMEM with 10% FBS and antibiotics. After 24 h cells were subjected to transfection with G6PD siRNA and control siRNA (scrambled sequence used as negative control) purchased from Santa Cruz Biotechnology (Santa Cruz, CA). Human G6PD siRNA (sc-60667) or Control siRNA (sc-307007) were used at different concentrations (1-5-10 nM) to identify the optimal concentration for a significant decrease of G6PD expression. For the optimal siRNA transfection was used the INTERFERin reagent (Polyplus trasfection). The G6PD expression inhibiting concentration (10 nM) was used for all silencing experiments. After 24 h of transfection siG6PD A549 cells were infected with PR8 for other 24 h, then cells were processed for the relative experiments.

### A549 NRF2 -/- Generation

NRF2 KO A549 cells were a kind gift of Christian K. Holm (Aarhus University). Briefly, the hNRF2 specific guide RNA sequence 5′-GCTGAAAACTTCGAGATATA-3′ was cloned into pLentiCRISPR/Cas9 V2 plasmid (a gift from Feng Zhang, Addgene #52961); the engineered vector was validated by Sanger sequencing and subsequently transfected in HEK293 cells, together with pMD.2G (Addgene), pRSV.Rev (Addgene) and pMDlg/p-RRE (Addgene), to produce infectious lentivirions. Knockout of NRF2 gene product was validated by immunoblotting.

### Real-Time PCR Assay

For G6PD, NRF2 and NP mRNA quantification, total RNA was isolated from cell lysates (Total RNA Purification Plus Kit, Norgen Biotek, Thorold, ON, Canada) and used as a template for generating cDNA (iScript cDNA Synthesis Kit, Bio-Rad, Milan, Italy). An aliquot of the cDNA was subjected to 40 cycles of RT-qPCR amplification (95°C, 10 sec; 60°C, 30 sec) using iQ SYBR Green Supermix and a LightCycler iQ 5 (Bio-Rad, Milan, Italy). The housekeeping gene ribosomal subunit 18S was used for normalization. Relative quantitative evaluation was performed by the comparative ΔΔCt method.

### Protein Extraction and Western Blot Analysis

Influenza virus-infected cells were lysed with RIPA lysis buffer [20 mM Tris–HCl pH 8, 150 mM NaCl, 1% Triton X-100, 0.1% sodium dodecyl sulfate (SDS), 0.5%] supplemented with phenylmethylsulfonyl fluoride, protease inhibitor mixture, and phosphatase inhibitor (Sigma- Aldrich, Milan, Italy) and total extract was analyzed by SDS-PAGE followed by western blotting with anti-G6PD (#12263 Cell Signaling), anti-NRF2 (#12721 Cell Signaling) anti-SIRT2 (MAB4358 R&D systems) and anti-influenza (AB1074 Merck Millipore, Darmstadt, Germany) antibodies. The nuclear and cytoplasmic separation was performed by using NE-PER™ extraction kit (#78833 ThermoFisher). The nuclear fraction was loaded on SDS-PAGE and NRF2 protein analyzed by western blot with anti-NRF2 antibody (#12721 Cell Signaling). HRP-linked anti-rabbit, anti-mouse and anti-goat (Jackson ImmunoResearch, Newmarket,UK) were used as secondary antibodies. The membranes were developed using Clarity Western ECL substrate (Bio-Rad, Hercules, CA, USA).

### G6PD Activity and GSH Production Assay

Equal amount (5x10^5^ cells) of A549 cells were seeded in a 6 multiwell plate and incubated for 24 h at 37°C. Then cells were infected with PR8 or mock-infected and after the following 24 h were lysed and the cellular extracts were analyzed for G6PD activity by using a colorimetric assay kit (#K757 Biovision). In the assay, glucose-6-phosphate is oxidized with the generation of a product which is utilized to convert a nearly colorless probe to an intensely colored product with an absorbance at 450 nm measured by a Multiskan EX Reader (Thermo Fisher Scientific, Monza, Italy). One unit is defined as the amount of enzyme that catalyzes the conversion of 1.0 μlmol of glucose-6-phosphate into 6-phosphoglucono-δ-lactone and generates 1.0 μmol of NAD+ to NADH per minute at 37°C. G6PD activity was extrapolated by a standard NADH curve. Data obtained from infected cells were expressed as percentage (%) compared to those from mock-infected cells (considered as 100%).

Intracellular glutathione (GSH) and oxidized forms of glutathione (GSSG) were measured in siG6PD and control siRNA cells by using the Glutathione Assay Kit (ADI-900-160 Enzo) following the manufacturer’s instructions, after deproteinization with metaphosphoric acid of the cell lysates. For GSSG quantification, an aliquot of deproteinized samples was first incubated with 2-vinylpyridine to derivatize GSH. Reduced GSH levels were obtained by differences between total GSH and GSSG and normalized to protein content of each sample determined by Bradford method (Bio-Rad, Hercules, CA, USA).

### In Cell Western Assay

A549 cells were seeded at a density of 2x10^4^ cells per well in a 96 multiwell plate. The monolayer was infected and after 24 h cells were washed with PBS twice and fixed with 50μl of 4% paraformaldehyde in PBS for 15 min at room temperature (RT), and then were permeabilized in 0.1% triton X-100 PBS for 5 min at RT. Following the incubation with Odyssey blocking buffer for 1 h at RT, cells were incubated with anti-G6PD (1:1000 sc-373886 Santa Cruz) or anti-influenza (AB1074 Merck Millipore, Darmstadt, Germany) antibodies. Afterward, labeled secondary IRDye 800 CW Goat Anti Mouse antibody (926-32210 LI-COR Biosciences, 1:3000 dilution in Odyssey Blocking buffer) and Cell Tag 700 Stain (926-41090, LI-COR Biosciences, 1:4000) were added to each well and after 1 h, cells were washed four times with PBS containing 0.1% Tween-20. Finally, the plate was scanned on the Odyssey Infrared Imager and the integrated intensities of fluorescence were determined by the LI-COR Image Studio software. The relative fluorescence unit (RFU) for each protein was normalized to Cell Tag and it was expressed as percentage compared to mock-infected cells (100%).

### Immunoprecipitation Assay

A549 cells were seeded at a density of 1.5x10^6^ cells per well and after 24 h the monolayer was infected with PR8 virus for 24 h. Cells were lysed with Lysis Buffer (10 mM Tris, 150 mM NaCl, and 0.25% NP-40, pH 7.4) and the total extract was preclearing with Prot A/G (sc 2003 Santa Cruz) over night at 4°C. After a centrifugation the supernatant was incubated with G6PD antibody (sc 373886 Santa Cruz) over night at 4°C and then immunoprecipitated for 2 h with Prot A/G. After 3 washes with the wash buffer (Tris 20 mM, NaCl 0.5M, Np40 0.25%, PMSF 1 mM) the samples were loaded on SDS-PAGE and analyzed by western-blot using anti-Acetyl Lisine antibody (#9441 Cell Signaling).

### AGK2 Treatment

A549 cells were seeded and treated with a SIRT2 inhibitor (AGK2, Santa Cruz) at different concentrations (from 3.5 to 30 μM) in order to select the optimal concentration to maintain cell viability. After 24 h, cells were infected with PR8 virus. Cells were lysed and then loaded in SDS-PAGE and analyzed by western blot by using anti-SIRT2 (MAB4358 R&D systems) and anti-G6PD (sc-373886 Santa Cruz) antibodies.

### iP300 Treatment

Confluent monolayer of A549 cells challenged with PR8 virus for 1 h at 37°C. After the viral adsorption the cells were washed with phosphate-buffered saline (PBS) and then incubated with iP300 molecule diluted in medium supplemented with 2% of FBS for 24 h. For the evaluation of the antiviral activity, the iP300 inhibitor was dissolved in DMSO and then diluted to the final concentrations of 25 μM in the cell culture medium. The highest DMSO concentration present in the culture medium was 0.2%. Control cells were treated with DMSO alone at the same concentration.

### Statistical Analysis

Statistical analyses were carried out using a two-tailed Student’s test. A *P* value < 0.05 was considered statistically significant. The data represents the mean of replicate experiments and the relative standard deviation (SD). Statistical analysis was performed by GraphPad Prism™ 6.0 software (GraphPad Software Inc., San Diego, California, USA).

## Results

### Influenza Virus Infection Reduces G6PD Expression and Activity

**Figure 1:**
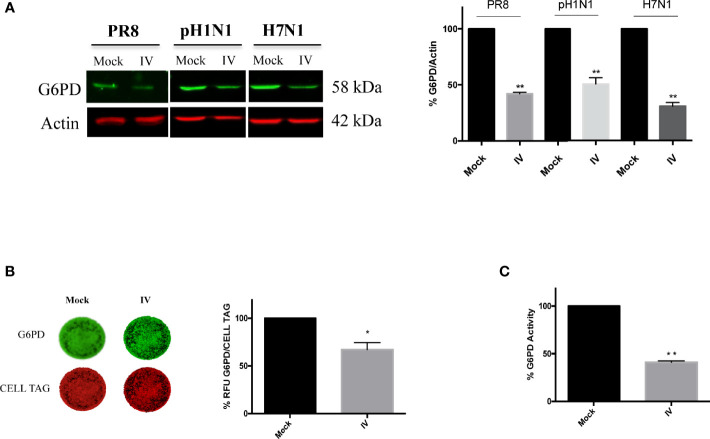
Influenza virus infection reduces G6PD expression and activity. **(A)** Western blot analysis of G6PD protein expression in A549 cells infected for 24 h with influenza virus A/PR/8/H1N1 (PR8), A/California/7/2009/H1N1 (pH1N1) or avian Parrot/Ulster/73 H7N1 (H7N1) influenza virus strains. Actin was used as loading control for the densitometry analysis of three independent experiments, each performed in duplicate (n = 6). Data are expressed as mean ± S.D. (**P < 0.001 *vs Mock*). **(B)** In Cell Western assay (ICW) of G6PD expression in Mock and influenza virus A/PR/8/H1N1 (IV) infected A549 cells. The images represent the expression of G6PD protein (green fluorescence) and of cell monolayer (red fluorescence - CELL TAG). The graph represents the percentage (%) of Relative Fluorescence Units (RFU) of G6PD expression normalized to CELL TAG of IV cells compared to Mock (considered as 100%). The analysis was performed measuring the RFU values of 4 different fields/well for each condition of the three experiments performed. Data are expressed as mean ± S.D. (*P < 0.05 *vs. Mock*). **(C)** G6PD activity evaluated in Mock and IV cells 24 h p.i. The values were normalized with those of the standard NADH curve, as described in materials and methods and the activity of the enzyme was reported as percentage (%) *vs* Mock. Data are the mean ± S.D. from three separate experiments each performed in triplicate (n = 9), (**P < 0.001 *vs. Mock*).

To examine the effect of influenza virus infection on G6PD expression and activity, A549 cells were infected with different strains of influenza virus and G6PD expression was monitored 24 h later (post infection, p.i.). As shown in **Figure 1A**, influenza virus A/PR/8/H1N1 (PR8) infection induced a significant decrease in G6PD protein expression by 60%. Similarly, in influenza virus A/California/7/2009/H1N1 (pH1N1) or avian Parrot/Ulster/73 H7N1 (H7N1) infected cells, G6PD protein was reduced by 50 and 70% respectively. These results were also confirmed through in cell western assay that detected a significant reduction of G6PD expression in A549 PR8 infected cells (IV) at 24 h after infection (**Figure 1B**). Consistently, under the same experimental conditions, G6PD enzyme activity was found reduced by 60% (**P < 0.001) (**Figure 1C**). These data indicate that influenza virus down-regulates both expression and enzymatic activity of G6PD.

### G6PD Knockdown on Infected Cells Exacerbates the Virus-Induced Oxidative Stress and Influenza Virus Replication

**Figure 2:**
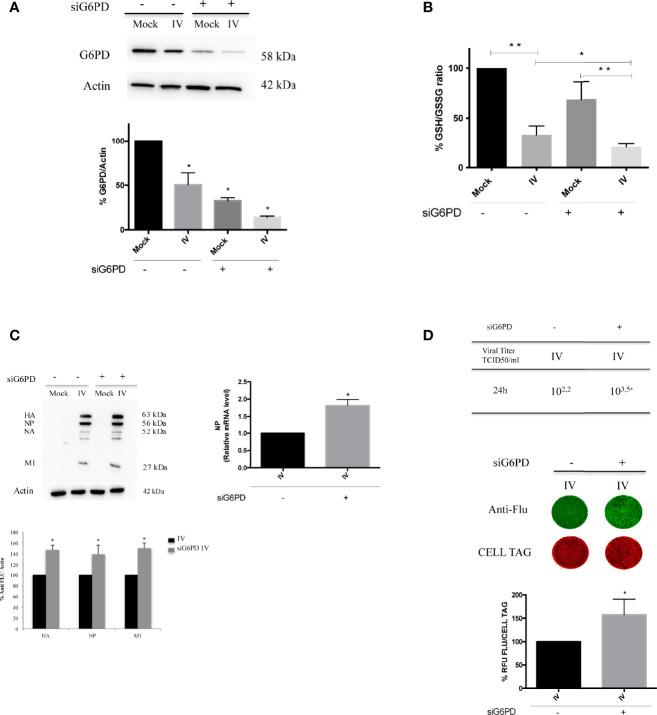
G6PD knockdown in infected cells exacerbates the virus-induced oxidative stress and influenza virus replication. **(A)** Western blot analysis of G6PD protein expression in A549 mock-infected control cells (Mock) and PR8-infected cells (IV) transfected with siRNA specific to G6PD (siG6PD) or with a control non-targeting siRNA (*P < 0.05 *vs. Mock control cells*). **(B)** Intracellular GSH/GSSG ratio measured after 24 h from infection by a colorimetric assay as described in methods and represented as percentage (%) respect to mock-infected control siRNA cells (considered 100%). The graph represents the mean ± S.D. obtained from three separate experiments, each performed in duplicate (n = 6) (*P < 0.05 *IV^s^* ^i^ *vs. IV*; **P < 0.001 I *vs. Mock and IV^si^ vs. Mock^si^*). **(C)** Western blot analysis of viral proteins: HA, Hemagglutinin; NP, Nucleoprotein; NA, Neuraminidase; M1, Matrix 1. The densitometry analysis was performed on three viral proteins normalized to Actin. Results are the mean ± S.D. obtained from three independent experiments, each performed in duplicate (n = 6) (*P < 0.05 *vs. IV*). In the right panel is represented the analysis of NP mRNA expression by RT-qPCR in infected control or silenced cells. Data are expressed as mean ± S.D, obtained from two independent experiments (*P < 0.05 *vs. IV*). **(D)** Viral load released in the supernatants of IV and IV^si^ A549 cells measured by TCID50 assay (*P < 0.05) (upper panel). Results are representative of one experiment of the three performed. In the bottom panel, ICW assay of the same supernatants. The images show viral proteins expression (green fluorescence) stained with anti-influenza antibody and cell monolayer (red fluorescence, CELL TAG). The graph shows the percentage (%) of RFU obtained from viral proteins normalized to CELL TAG of IV^si^ cells compared to IV (considered as 100%). The analysis was performed measuring the RFU values of 4 different fields/well for each condition from the three experiments performed. Data are expressed as mean ± S.D (*P < 0.05 *vs. IV*).

As stated above, basal G6PD activity contributes to the regeneration of NADPH that is required for the maintenance of the cellular GSH pool (Salvemini et al., 1999); indeed, cells deficient in G6PD are incapable of regenerating sufficient NADPH following cellular stress (Yang et al., 2019). Therefore, we evaluated the effect of G6PD deficiency on the GSH production during influenza virus infection using siRNA mediated silencing of G6PD expression (10 nM of siRNA for 48 h) that silenced G6PD expression by 70% (**Figure 2A**). Scrambled sequence was used as negative control (control siRNA), as described in Materials and Methods.

As shown in **Figure 2B**, G6PD silencing in mock-infected cells induced a decrease of GSH/GSSG ratio (~40% decrease) compared to mock control siRNA cells. As expected, we found that the GSH/GSSG ratio was decreased in both infected conditions (~65% compared to mock-infected counterparts, **P < 0.001). Interestingly, in siG6PD infected cells this ratio was significantly lower with respect to control siRNA infected cells (*P < 0.05). These data suggest that G6PD impairment contributes to the reduction in GSH levels during infection.

G6PD silencing was paralleled by a higher viral replication at 24 h, as determined by densitometry quantification of hemagglutinin (HA), nucleoprotein (NP) and matrix protein type 1 (M1), (50% of increase) (*P < 0.05) (**Figure 2C**, left panel). Accordingly, the NP mRNA expression was found increased in siG6PD infected cells compared to control ones (**Figure 2C**, right panel) (*P < 0.05). The TCID50 assay also demonstrated that the viral titer was 1.3 log higher in siRNA silenced cells (**Figure 2D**, upper panel) (*P < 0.05). Similarly, ICW analysis showed that cells silenced for G6PD displayed higher expression (~60%) of viral proteins compared to infected control cells (*P < 0.05) (**Figure 2D**, bottom panel).

### G6PD Protein Level Decrease Is Related to a Drop in the NRF2-Mediated Antioxidant Response During the Infection

To investigate the mechanism through which influenza virus induces G6PD protein decrease, we analyzed the expression of total NRF2 protein, that is involved in the antioxidant response activation, as well as in G6PD transcription (Ramezani et al., 2018).

**Figure 3:**
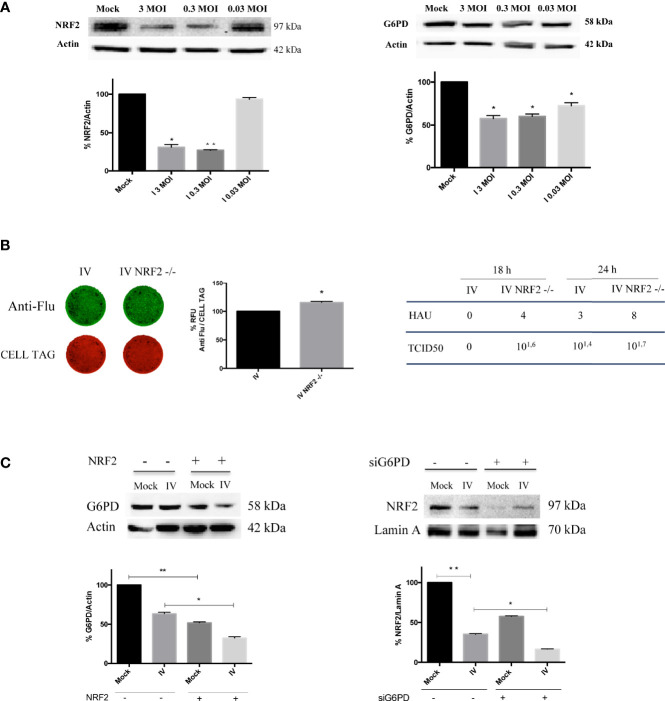
Downregulation of NRF2-mediated antioxidant response improves influenza virus infection. **(A)** Western blot analysis of NRF2 and G6PD protein expression in A549 cells infected with different MOI (3, 0.3 and 0.03) of PR8 for 24 h. Actin was used as loading control. Data are the mean ± S.D. of two independent experiments, each condition run in duplicate (n = 4) (*P < 0.05 and **P < 0.001 *vs. Mock*). **(B)** ICW of viral proteins expression in A549 wild-type (IV) and A549 NRF2 -/- cells (IV NRF2 -/-) (left panel). The images show viral proteins expression (green fluorescence, anti-Flu) and cell monolayer (red fluorescence, CELL TAG). The graph represents the percentage (%) of RFU obtained from viral proteins normalized to CELL TAG. The analysis was performed measuring the RFU values of 4 different fields/well for each condition from the three experiments performed. Data are expressed as mean ± S.D. (*P < 0.05 *vs. IV*). Viral titer measured in the supernatants of A549 wild-type (IV) and A549 NRF2 -/- cells (IV NRF2 -/-) by Hemagglutination (HAU) and TCID50 assays (right panel). **(C)** Western blot analysis of G6PD expression in NRF2 -/- or wild type A549 cells infected with PR8 virus (IV conditions) for 24 h. Densitometry analysis of data obtained from two experiments performed; the values are expressed as mean ± S.D. (**P < 0.001, *Mock vs. Mock -/-*; *P < 0.05, *IV vs. IV -/-*). In the right panel is represented the analysis of NRF2 protein expression in the nuclear extracts of silenced G6PD or normal cells infected or not with PR8 virus (IV), left panel. Lamin was used as loading control. Densitometry analysis of data obtained from the two experiments performed; the values are expressed as mean ± S.D. (**P < 0.001, IV *vs. Mock*; *P < 0.05, *IV^si^ vs. IV*).

We found that NRF2 expression was strongly reduced in cells infected with 3 and 0.3 MOI; similarly, G6PD expression showed a reduction at the same conditions (**Figure 3A**) suggesting a possible involvement of NRF2-mediated antioxidant response in regulating G6PD during IV infection. The virus-mediated decrease of G6PD and NRF2 protein expression was also confirmed in three others cell lines, MDCK, HEK293 and BEAS-2B known to be highly permissive to influenza virus infection (Nencioni et al., 2003; Amatore et al., 2015) (**Supplementary Figure 1**).

To verify the hypothesis that the down-modulation of the antioxidant pathway could promote virus replication in a manner similar to G6PD depletion (**Figure 2C**), viral proteins were measured in A549 cells knocked out for NRF2 using CRISPR/Cas9. As shown in **Figure 3B** (left panel), the virus proteins expression stained with anti-Flu antibodies was increased in NRF2-/- cells (IV NRF2-/-) compared to influenza virus infected wild type A549 cells (IV), suggesting that the absence of the NRF2-mediated antioxidant response is useful for promoting viral replication. Accordingly, influenza virus titer measured in NRF2 -/- cells by HAU and TCID50 assays at 18 and 24 h after infection, was higher compared to IV (**Figure 3B**, right panel).

Moreover, G6PD expression was further reduced in NRF2-/- cells compared to infected wild type cells (**Figure 3C**, left panel), indicating a correlation between the modulation of the antioxidant response and G6PD protein decrease.

Interestingly, the inhibition of G6PD or the interference with the pentose phosphate pathway hampers the NRF2-mediated detoxification of cells (Heiss et al., 2013). Based on this evidence, we examined the NRF2 activation in G6PD silenced conditions by evaluating the NRF2 nuclear localization. As expected from the results obtained in **Figure 3A**, virus infection decreased NRF2 nuclear translocation by 60%, compared to mock-infected cells (**Figure 3C**, right panel). Moreover, the absence of G6PD negatively affected NRF2 pathway. Indeed, in siG6PD cells the NRF2 protein was further decreased compared to control cells, in both mock-infected and virus-infected cells (**Figure 3C**, right panel).

**Figure 4:**
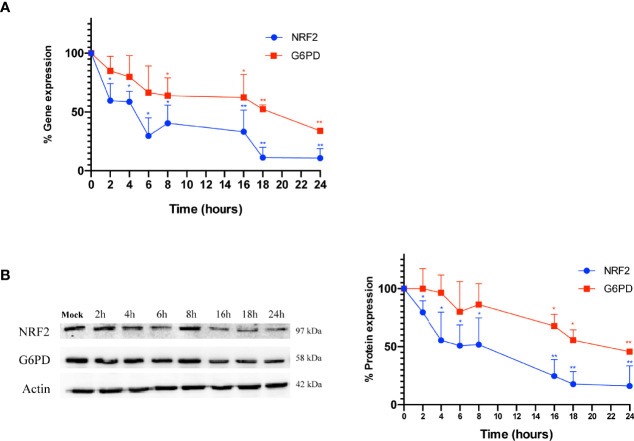
G6PD protein reduction is mediated by a drop in the NRF2-mediated antioxidant response during the infection. **(A)** RT-qPCR analysis of NRF2 and G6PD gene expression during a time course (from 2 to 24 h p.i.) of PR8 virus infection. The analysis of NRF2 (blu line) and G6PD (red line) mRNA levels normalized to those of 18S (control) was performed in two independent experiments, each performed in duplicate (n = 4). Values are expressed as the mean ± S.D. (*P < 0.05 *vs. 0* h*, Mock-infected cells*). **(B)** Western blot analysis of NRF2 (blu line) and G6PD (red line) protein synthesis during a time course (from 2 to 24 h p.i.) of PR8 infection. Actin was used as loading control. The densitometry analysis of values was obtained from two independent experiments, each sample run in duplicate. Data are the mean ± S.D. (n = 4) (*P < 0.05 and **P < 0.001 vs. Mock).

Next, to evaluate whether the down-regulation of G6PD expression in infected cells is mediated by a reduction of its transcription factor, both NRF2 and G6PD mRNA and protein levels were analyzed at different times (2-24 h) of infection. As shown in **Figure 4A**, the expression of NRF2 mRNA decreased within hours of influenza virus infection, with the lowest levels observed at 18 h and 24 h (**P < 0.001); NRF2 protein levels displayed similar kinetics (**Figure 4B**). Expression of G6PD mRNA was found to decrease at 8 h after infection until 24 h (*P < 0.05) (**Figure 4A**), while G6PD protein levels significantly decreased beginning at 16 h p.i (*P < 0.05) (**Figure 4B**). These results demonstrate that a decrease in the NRF2-mediated antioxidant response, as result of NRF2 inhibition, contributed to the decrease in G6PD expression after infection.

### G6PD Acetylation Increases With the Infection and Correlates With A Down-Modulation of SIRT2 Deacetylase

**Figure 5:**
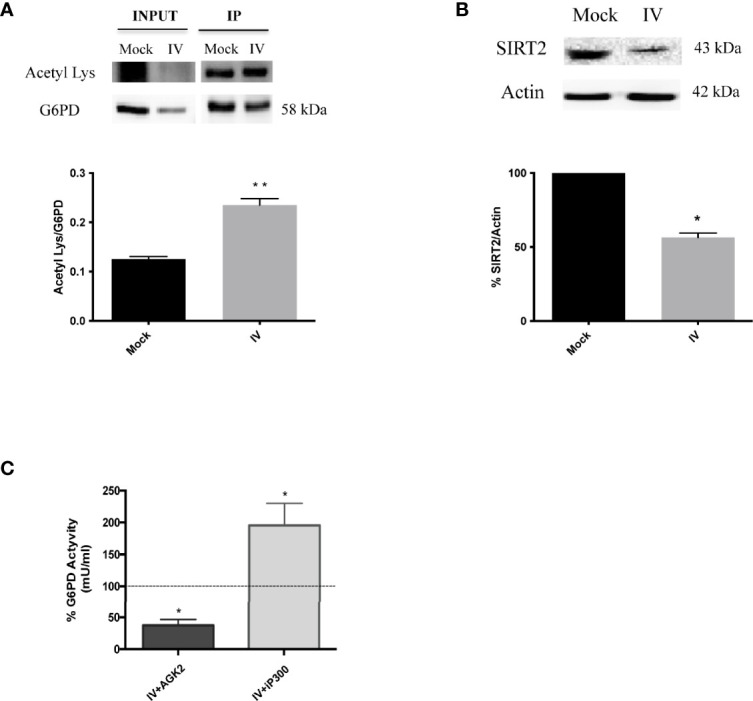
G6PD is more acetylated after the infection and it correlates with a downmodulation of SIRT2 deacetylase. **(A)** Immunoprecipitation assay of G6PD protein in mock-infected control (Mock) and PR8 virus-infected A549 cells (IV) 24 h p.i. Blot is representative of two independent experiments and shows the G6PD expression of total cell lysate (INPUT) and in the immunoprecipitated (IP) samples. Densitometry analysis in the graph shows the ratio of Acetylated Lysine on the immunoprecipitated protein (Acetyl Lys/G6PD) of two independent experiments performed in duplicate (n = 4). Data are the mean ± SD (**P < 0.001 *vs. Mock*). **(B)** Western blot analysis of SIRT2 protein expression in A549 cells infected with PR8 virus for 24 h. The graphs represent the densitometry analysis of SIRT2 protein expression. Actin was used as loading control for the analysis of three experimental replicates (n = 3). Data are the mean ± SD (*P < 0.05 vs. Mock). **(C)** G6PD activity in A549 infected cells treated with AGK2 inhibitor (IV+AGK2) or with iP300 (IV+iP300). The values were normalized with those of the standard NADH curve as described in materials and methods. G6PD activity is expressed as percentage (%) with respect to that obtained from untreated-infected cells which is indicated by the dashed black line (considered as 100%). Data are represented as mean ± S.D. obtained from three experiments performed (*P < 0.05 vs. IV untreated cells).

Influenza virus infection resulted in a decrease of G6PD expression at both the protein and activity levels. To determine whether inhibition of G6PD activity by influenza virus infection involved post-translational mechanisms other than antioxidant gene regulation, we next evaluated the role of SIRT2 in regulating G6PD dimer formation, based on previous studies demonstrating that deacetylation of G6PD at Lys403 stimulated dimer formation and consequently the G6PD activity (Wang et al., 2014). G6PD, immunoprecipitated from influenza infected or mock-infected cells and probed with anti-acetyl lysine antibody, showed a higher level of lysine residue acetylation in influenza virus-infected cells (**P < 0.001) (**Figure 5A**), thus indicating that G6PD was modulated by post-translational acetylation/deacetylation after influenza virus infection. Furthermore, SIRT2 deacetylase expression was shown to be significantly reduced after virus infection (*P < 0.05) (**Figure 5B**). Using a selective inhibitor of SIRT2 deacetylase (AGK2) (Outeiro et al., 2007), G6PD activity was further decreased in A549 cells compared to infected untreated cells (*P < 0.05) (**Figure 5C**). Since G6PD levels were also reduced in mock-infected treated cells (data not shown), our data suggest a direct correlation between SIRT2 and G6PD activity during infection. To determine if G6PD activity could be increased in infected cells by restoring SIRT2 activity, A549 cells were treated with C646, an inhibitor of acetyltransferase p300 known to down-regulate the activity of SIRT2 (Han et al., 2008). The treatment with C646 for 24 h p.i. by restoring SIRT2 functionality increased G6PD activity in influenza virus infected cells (**Figure 5C**), as well as in mock-infected cells (data not shown). Together, these results establish a close relationship between the acetylation/deacetylation of G6PD and its activity in influenza virus infected cells.

### The Re-Activation of SIRT2 and G6PD Rescues the Antioxidant Response and Decreases Viral Titer

**Figure 6:**
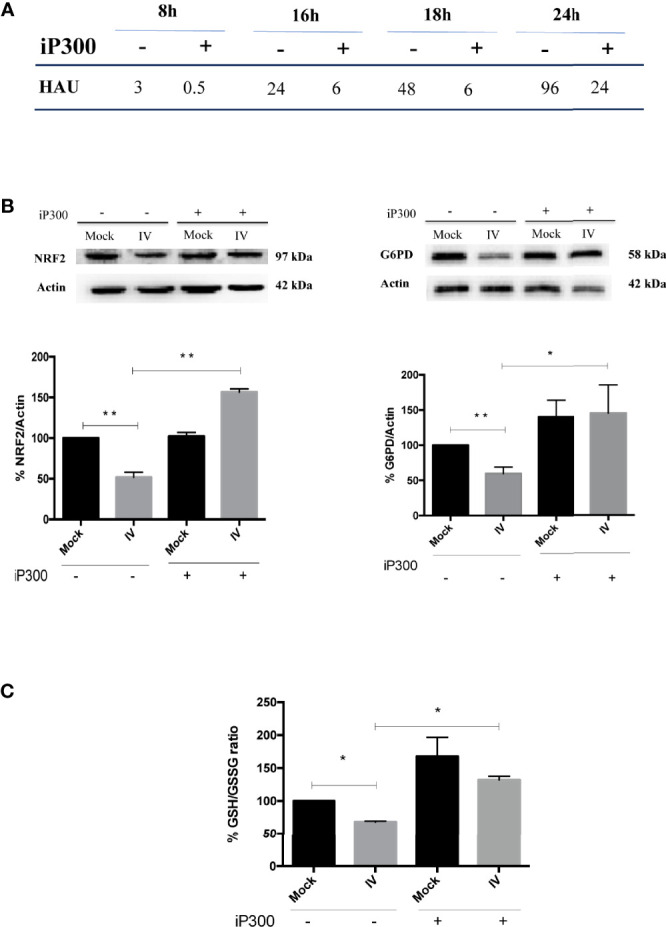
Treatment with P300 inhibitor rescues the antioxidant response and decreases viral titer. **(A)** Viral titer measured at different time after infection (8-24 h) in the supernatants of infected (IV) and infected cells treated with 25 mM C646 (IV+iP300) cells by Hemagglutination assay (HAU). **(B)** Western Blot analysis of NRF2 (*left panel*) and G6PD (*right panel*) proteins in A549 cells treated with iP300 (C646). Actin was used as loading control. Densitometry analysis of two technical replicates of one representative experiment. Data are the mean ± S.D. (*P < 0.05 vs. Mock or IV not treated cells; **P < 0.001 vs. not treated cells). **(C)** The intracellular GSH/GSSG ratio measured in the same conditions using a colorimetric assay. Data are the mean ± S.D. of three technical replicates of one representative experiment (*P < *0.05 vs. Mock and IV not treated cells*).

To confirm the role of SIRT2 and G6PD in regulating influenza virus replication, viral titer was measured in A549 cells treated with the inhibitor of p300 compared with untreated cells. As shown in **Figure 6A**, viral titers measured at different times (8-24 h) from infection decreased after iP300 treatment. Moreover, in both infected and mock-infected cells treated with inhibitor, levels of NRF2 and G6PD proteins (**Figure 6B**), as well as intracellular GSH/GSSG ratio (**Figure 6C**), increased in comparison to untreated cells. The results are consistent with previous studies demonstrating the capacity of SIRT2 to induce NRF2 activation and GSH production (Cao et al., 2016). Moreover, the reduction in influenza virus titer by restoration of the antioxidant pathway is in line with our previous data reporting that reducing conditions play a key role in controlling viral replication (Sgarbanti et al., 2011; Celestino et al., 2018; Di Sotto et al., 2018; Amatore et al., 2019). Altogether, these results identify an additional mechanism used by influenza to down-modulate G6PD enzyme at the level of deacetylation/acetylation. Virus-induced down-regulation of G6PD occurred both at the transcriptional level *via* NRF2 modulation, and at the level of SIRT2-dependent deacetylation.

## Discussion

**Figure 7:**
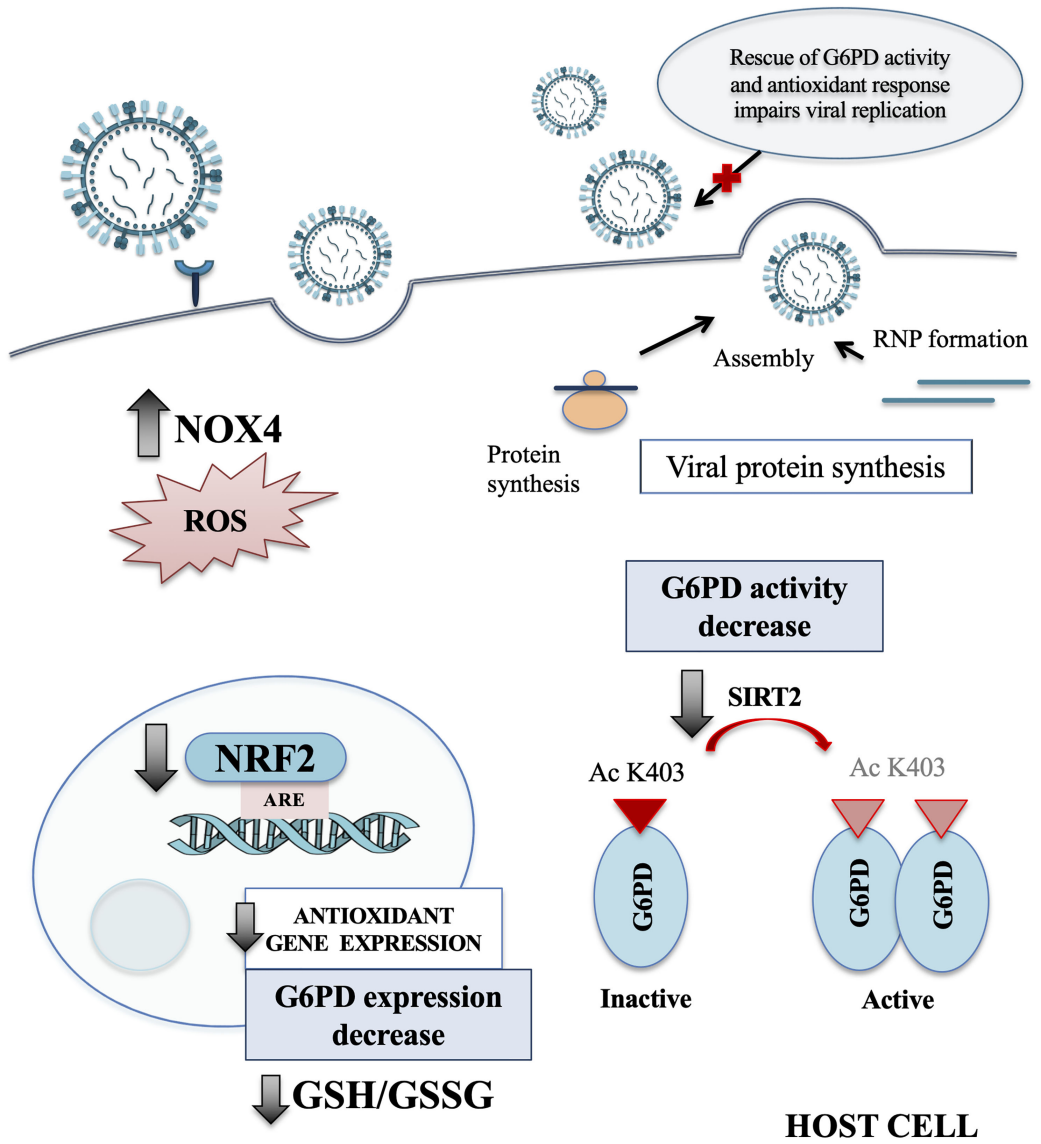
Influenza virus infection affects the host antioxidant response. Schematic representation of the main redox-related pathways modulated by influenza virus infection. NRF2 protein is present at lower level into the nucleus of host infected cells, leading to a drop of the antioxidant gene expression, including G6PD. The reduction of SIRT2 expression affects the activation of G6PD enzyme that in turn leads to GSH/GSSG ratio decrease. Moreover, the infection induces oxidative stress condition also by increasing the NOX4-mediated ROS production. Finally, we observed that the rescue of NRF2 and G6PD expression impairs influenza virus replication. NOX4, nicotinamide adenine dinucleotide phosphate oxidase 4; ROS, reactive oxygen species; NRF2, nuclear factor erythroid 2-related factor 2; ARE, antioxidant response elements; G6PD, glucose-6-phosphate dehydrogenase; GSH, glutathione; GSSG, oxidized form of GSH; SIRT2, sirtuin 2; Ac K403, acetylated lysine 403; RNP, ribonucleoprotein.

In the present study, we identify a novel mechanism used by influenza virus to tip the intracellular redox balance towards increased oxidative stress for its own replicative advantage (**Figure 7**). Specifically, we demonstrated that the virus down-modulates G6PD enzyme at protein and activity levels (**Figure 1**). Importantly, G6PD plays a pivotal role in restoration of NADPH-dependent GSH levels (Lu, 2009; Yang et al., 2019). Previous data, including ours, demonstrated that influenza virus infection caused an imbalance of the intracellular redox state, resulting in elevated oxidative stress, by increasing ROS production and depleting GSH (Cai et al., 2003; Nencioni et al., 2003; Sgarbanti et al., 2011; To et al., 2014; Amatore et al., 2015; Celestino et al., 2018; Checconi et al., 2020). Here we further demonstrated that the decrease of G6PD expression was accompanied by an increase in oxidative stress and virus replication (**Figure 2**).

Several viruses induce oxidative stress conditions by modulating the NRF2 antioxidant response pathway (Ramezani et al., 2018); for example, Dengue virus induces NRF2 degradation, leading to increased ROS production and oxidative stress, as well as suppression of the host antiviral response (Ferrari et al., 2020). Modulation of NRF2 activity by influenza virus has also been previously demonstrated, although conflicting data have been reported. On the one hand, influenza virus-induced oxidative stress activated the NRF2 antioxidant network, which in turn protected infected cells against virus-induced cellular injury (Kosmider et al., 2012); on the other hand, proteomic analysis identified a phosphorylated form of NRF2 that was not imported into the nucleus after the infection with highly pathogenic influenza virus strains (Simon et al., 2015), suggesting that influenza virus inhibited NRF2 pathway activation. Furthermore, overexpression of NRF2 in alveolar epithelial cells decreased virus replication and the associated oxidative stress, whereas NRF2 knockdown conferred increased susceptibility to influenza A virus infection (Kesic et al., 2011). Consistent with these latter observations, we also found that NRF2 expression was reduced after the infection (**Figure 3A**), leading to increased virus multiplication (**Figure 3B**), supporting the view that the influenza virus disabled the NRF2 antioxidant response to facilitate its own replication.

Literature evidence reported that in lung adenocarcinoma epithelial cells, mutations in Keap1 genomic locus result in the increase of NRF2 pathway activation (Singh et al., 2006). Accordingly, in our model we found a higher expression of NRF2 protein in the total extract of A549 uninfected cells. The fact that these cells contain high NRF2 expression at physiological level, further supports the observed NRF2 protein decrease in infected cells and confirms the regulation of host antioxidant response by viral infection (**Figure 3A**). Indeed, we observed a reduction of both NRF2 and G6PD expression also in non-tumor cell lines such as MDCK, HEK293 and BEAS-2B (**Supplementary Figure 1**) indicating the modulation of NRF2 pathway also in normal infected cells.

Accordingly, we observed that NRF2 nuclear localization was decreased after the infection (**Figure 3C**, right panel). Moreover, NRF2 activity was further decreased in cells silenced for G6PD, consistent with those studies demonstrating that a functional pentose phosphate pathway is linked to the NRF2-mediated antioxidant response (Heiss et al., 2013). Interestingly, influenza virus inhibited NRF2 expression within 2 h of infection and induced a subsequent inhibition of downstream antioxidant genes, including G6PD itself (**Figures 4A, B**). Overall, these results highlight the regulation of G6PD protein during the infection by NRF2 and underscore the interrelationship between glucose metabolism and susceptibility to viral infections during glucose metabolic alterations, supporting the central role of G6PD pathway modulation.

Along with regulation by redox balance, G6PD activity is also modulated by post-translational acetylation/deacetylation; proteomic acetylome studies revealed that G6PD protein was acetylated at multiple lysine residues, among which Lys403 was a key regulatory site (Lundby et al., 2012; Lemos et al., 2017). We also detected lysine hyperacetylation of G6PD in influenza virus-infected cells, coupled with the inhibition of the SIRT2 deacetylase (**Figures 5A, B**). Although G6PD protein levels were significantly reduced after influenza virus infection, G6PD protein was highly acetylated, probably due to the decreased activity of SIRT2.

The role of sirtuins in controlling viral replication has been evaluated for different DNA or RNA viruses. Indeed, the knockdown of individual sirtuins, including SIRT2, has been shown to increase the influenza virus replication (Koyuncu et al., 2014). Moreover, treatment with sirtuin modulators including resveratrol and its derivatives have been shown to control different respiratory viral infections (Palamara et al., 2005; De Angelis et al., 2021; Pasquereau et al., 2021). In our model, by restoring the deacetylation activity of SIRT2 using an inhibitor of P300 acetyltransferase (C646), viral replication was reduced (**Figure 6A**). The inhibitory effect was linked to increased G6PD activity (**Figure 5C**), NRF2-mediated antioxidant response and intracellular GSH content (**Figures 6B, C**).

Even though some authors report that p300 controls NRF2 stability (Ganner et al., 2020), others show that SIRT2 stimulation induces NRF2-regulated gene transcription, including those involved in GSH synthesis and pentose phosphate pathway (Cao et al., 2016). Accordingly, our data demonstrate that iP300, by activating SIRT2, was able to upregulate the NRF2-mediated antioxidant response.

Zhao D. et al. (Zhao et al., 2015) showed that C646 modulated several host genes during viral infection, including G6PD. Thus, although we cannot exclude off-target effects, our data suggest that p300/SIRT2 pathway may contribute to the regulation of G6PD activity during IV infection and suggest G6PD as a potential target to control the redox cell environment in virus-infected cells.

Several studies, including epidemiological ones, have likewise established a correlation between NRF2-mediated antioxidant response and viral replication rate (Ramezani et al., 2018; Anticoli et al., 2019; Shytaj et al., 2021). Interestingly, individuals with G6PD deficiency showed a higher prevalence of Hepatitis A and Hepatitis E virus infections than normal individuals (Miri-Aliabad et al., 2017; Ahmad et al., 2018), suggesting that increased oxidative stress in the absence of G6PD determined susceptibility to infection. Furthermore, G6PD-deficient cells supported EV71 replication more efficiently than wild type cells and displayed increased cytopathic effect and loss of viability (Ho et al., 2008). Likewise, G6PD knockdown increased the susceptibility to HCoV 229E infection (Wu et al., 2008), suggesting a correlation between G6PD deficiency and susceptibility to SARS-CoV-2 infection (Buinitskaya et al., 2020; Jain et al., 2020; Vick, 2020). Our data on influenza virus support this view and suggest that G6PD-deficiency contributes to virus susceptibility *via* increased oxidative stress. Moreover, given the importance of G6PD enzyme in glucose metabolism and its role in metabolic dysfunctions including diabetes, obesity and insulin resistance (Xu et al., 2005; Ham et al., 2013; Park et al., 2017; Wang et al., 2019), we can speculate that the virus-induced down-modulation of G6PD may contribute to the higher susceptibility to influenza virus infection in metabolic disorders (Hulme et al., 2017; Huo et al., 2017; Honce and Schultz-Cherry, 2019).

In conclusion, our data demonstrate that influenza virus exerts a fine regulation of G6PD activity by inducing high levels of oxidative stress that would be beneficial to virus replication. We observed a dual mechanism by which the virus down-modulates G6PD at the level of down-regulation of NRF2 expression and inhibition of SIRT2 activity, although further studies are in progress to identify whether specific viral proteins drive this process.

These results highlight the expression and activity of these redox-sensitive enzymes as potential targets for pharmaceutical strategies aimed at controlling virus replication especially during metabolic disorders.

## Data Availability Statement

The raw data supporting the conclusions of this article will be made available by the authors, without undue reservation.

## Author Contributions

MDA designed the study, contributed to the execution of the experiments, analysis and interpretation of data and drafted the paper. DA, PC, AZ, and AF participated in the interpretation of data. MM, JH, GDC, and ATP reviewed the manuscript. LN designed, supervised the research and reviewed the manuscript. All authors contributed to the article and approved the submitted version.

## Funding

This work was partially supported by: Fondazione Cenci Bolognetti Istituto Pasteur Italia grants (LN) and Ateneo grants (LN), MIUR PRIN: 2017BMK8JR006 (LN); 2017KM79NN (PC); 2020KSY3KL (ATP) grants.

## Conflict of Interest

The authors declare that the research was conducted in the absence of any commercial or financial relationships that could be construed as a potential conflict of interest.

## Publisher’s Note

All claims expressed in this article are solely those of the authors and do not necessarily represent those of their affiliated organizations, or those of the publisher, the editors and the reviewers. Any product that may be evaluated in this article, or claim that may be made by its manufacturer, is not guaranteed or endorsed by the publisher.

## Supplementary Material

Supplementary Figure 1 Influenza virus infection induces G6PD and NRF2 expression decrease in permissive cell lines. **(A)** Western blot analysis of G6PD protein expression in MDCK cells infected for 24 h with influenza virus A/PR/8/H1N1 (IV). Actin was used as loading control for the densitometry analysis of two independent experiments. Data are expressed as mean ± S.D *(*P < 0.001 vs Mock*). **(B)** Western blot analysis of G6PD and NRF2 protein expression in HEK293 cells infected for 24 h with influenza virus A/PR/8/H1N1 (IV). Actin was used as loading control for the densitometry analysis of two independent experiments. In the right panel, are represented the gene expression analysis of both NRF2 and G6PD during time course experiments (2-24 h). Data are expressed as mean ± S.D. (**P < 0.05 and **P < 0.001 vs Mock*). **(C)** G6PD and NRF2 protein expression in BEAS-2B cell line after 24h of IV PR8 infection. Actin was used as loading control for the densitometry analysis of two independent experiments. In the right panel, are represented the gene expression analysis of both NRF2 and G6PD during time course experiments (2-24 h). Data are expressed as mean ± S.D. (**P < 0.05 vs Mock*).

## References

[B1] Ahmad B. S. Ahmad A. Jamil S. Abubakar Mohsin Ehsanullah S. A. Munir A. (2018). Severe Haemolysis and Renal Failure Precipitated by Hepatitis E Virus in G6PD Deficient Patient: A Case Report. J. Pak. Med. Assoc. 68 (9), 1397–1399. 30317274

[B2] Amatore D. Celestino I. Brundu S. Galluzzi L. Coluccio P. Checconi P.. (2019). Glutathione Increase by the N-Butanoyl Glutathione Derivative (GSH-C4) Inhibits Viral Replication and Induces a Predominant Th1 Immune Profile in Old Mice Infected With Influenza Virus. FASEB Bioadv. 1 (5), 296–305. doi: 10.1096/fba.2018-00066 32123833 PMC6996388

[B3] Amatore D. Sgarbanti R. Aquilano K. Baldelli S. Limongi D. Civitelli L.. (2015). Influenza Virus Replication in Lung Epithelial Cells Depends on Redox-Sensitive Pathways Activated by NOX4-Derived ROS. Cell. Microbiol. 17 (1), 131–145. doi: 10.1111/cmi.12343 25154738 PMC4311438

[B4] Anticoli S. Amatore D. Matarrese P. De Angelis M. Palamara A. T. Nencioni L.. (2019). Counteraction of HCV-Induced Oxidative Stress Concurs to Establish Chronic Infection in Liver Cell Cultures. Oxid. Med. Cell. Longev. 2019, 6452390. doi: 10.1155/2019/6452390 30906503 PMC6393922

[B5] Baird L. Yamamoto M. (2020). The Molecular Mechanisms Regulating the KEAP1-Nrf2 Pathway. Mol. Cell. Biol. 40 (13), e00099–e00020. doi: 10.1128/MCB.00099-20 32284348 PMC7296212

[B6] Buinitskaya Y. Gurinovich R. Wlodaver C. G. Kastsiuchenka S. (2020). Centrality of G6PD in COVID-19: The Biochemical Rationale and Clinical Implications. Front. Med. 7, 584112. doi: 10.3389/fmed.2020.584112 PMC7643021 33195336

[B7] Cai J. Chen Y. Seth S. Furukawa S. Compans R. W. Jones D. P. (2003). Inhibition of Influenza Infection by Glutathione. Free Radic. Biol. Med. 34 (7), 928–936. doi: 10.1016/s0891-5849(03)00023-6 12654482

[B8] Cao W. Hong Y. Chen H. Wu F. Wei X. Ying W. (2016). SIRT2 Mediates NADH-Induced Increases in Nrf2, GCL, and Glutathione by Modulating Akt Phosphorylation in PC12 Cells. FEBS Lett. 590 (14), 2241–2255. doi: 10.1002/1873-3468.12236 27264719

[B9] Celestino I. Checconi P. Amatore D. De Angelis M. Coluccio P. Dattilo R.. (2018). Differential Redox State Contributes to Sex Disparities in the Response to Influenza Virus Infection in Male and Female Mice. Front. Immunol. 9, 1747. doi: 10.3389/fimmu.2018.01747 30105026 PMC6077261

[B10] Checconi P. De Angelis M. Marcocci M. E. Fraternale A. Magnani M. Palamara A. T.. (2020). Redox-Modulating Agents in the Treatment of Viral Infections. Int. J. Mol. Sci. 21 (11), 4084. doi: 10.3390/ijms21114084 PMC7312898 32521619

[B11] Choudhary C. Kumar C. Gnad F. Nielsen M. L. Rehman M. Walther T. C.. (2009). Lysine Acetylation Targets Protein Complexes and Co-Regulates Major Cellular Functions. Science 325 (5942), 834–840. doi: 10.1126/science.1175371 19608861

[B12] De Angelis M. Della-Morte D. Buttinelli G. Di Martino A. Pacifici F. Checconi P.. (2021). Protective Role of Combined Polyphenols and Micronutrients Against Influenza A Virus and SARS-CoV-2 Infection *In Vitro* . Biomedicines 9 (11), 1721. doi: 10.3390/biomedicines9111721 34829949 PMC8615651

[B13] Dickinson D. A. Forman H. J. (2002). Cellular Glutathione and Thiols Metabolism. Biochem. Pharmacol. 64 (5-6), 1019–1026. doi: 10.1016/s0006-2952(02)01172-3 12213601

[B14] Di Sotto A. Checconi P. Celestino I. Locatelli M. Carissimi S. De Angelis M.. (2018). Antiviral and Antioxidant Activity of a Hydroalcoholic Extract From Humulus Lupulus L. Oxid. Med. Cell. Longev. 2018, 5919237. doi: 10.1155/2018/5919237 30140367 PMC6081516

[B15] Ferrari M. Zevini A. Palermo E. Muscolini M. Alexandridi M. Etna M. P.. (2020). Dengue Virus Targets Nrf2 for NS2B3-Mediated Degradation Leading to Enhanced Oxidative Stress and Viral Replication. J. Virol. 94 (24), e01551–e01520. doi: 10.1128/JVI.01551-20 32999020 PMC7925186

[B16] Forman H. J. Zhang H. Rinna A. (2009). Glutathione: Overview of its Protective Roles, Measurement, and Biosynthesis. Mol. Aspects. Med. 30 (1-2), 1–12. doi: 10.1016/j.mam.2008.08.006 18796312 PMC2696075

[B17] Ganner A. Pfeiffer Z. C. Wingendorf L. Kreis S. Klein M. Walz G.. (2020). The Acetyltransferase P300 Regulates Nrf2 Stability and Localization. Biochem. Biophys. Res. Commun. 524 (4), 895–902. doi: 10.1016/j.bbrc.2020.02.006 32057361

[B18] Ham M. Lee J. W. Choi A. H. Jang H. Choi G. Park J.. (2013). Macrophage Glucose-6-Phosphate Dehydrogenase Stimulates Proinflammatory Responses With Oxidative Stress. Mol. Cell. Biol. 33 (12), 2425–2435. doi: 10.1128/MCB.01260-12 23572562 PMC3700093

[B19] Han Y. Jin Y. H. Kim Y. J. Kang B. Y. Choi H. J. Kim D. W.. (2008). Acetylation of Sirt2 by P300 Attenuates its Deacetylase Activity. Biochem. Biophys. Res. Commun. 375 (4), 576–580. doi: 10.1016/j.bbrc.2008.08.042 18722353

[B20] Heiss E. H. Schachner D. Zimmermann K. Dirsch V. M. (2013). Glucose Availability Is a Decisive Factor for Nrf2-Mediated Gene Expression. Redox Biol. 1 (1), 359–365. doi: 10.1016/j.redox.2013.06.001 24024172 PMC3757705

[B21] Ho H. Y. Cheng M. L. Weng S. F. Chang L. Yeh T. T. Shih S. R.. (2008). Glucose-6-Phosphate Dehydrogenase Deficiency Enhances Enterovirus 71 Infection. J. Gen. Virol. 89 (Pt 9), 2080–2089. doi: 10.1099/vir.0.2008/001404-0 18753216

[B22] Honce R. Schultz-Cherry S. (2019). Impact of Obesity on Influenza A Virus Pathogenesis, Immune Response, and Evolution. Front. Immunol. 10, 1071. doi: 10.3389/fimmu.2019.01071 31134099 PMC6523028

[B23] Hulme K. D. Gallo L. A. Short K. R. (2017). Influenza Virus and Glycemic Variability in Diabetes: A Killer Combination? Front. Microbiol. 8, 861. doi: 10.3389/fmicb.2017.00861 28588558 PMC5438975

[B24] Huo C. Zhang S. Zhang S. Wang M. Qi P. Xiao J.. (2017). Mice With Type 1 Diabetes Exhibit Increased Susceptibility to Influenza A Virus. Microb. Pathog. 113, 233–241. doi: 10.1016/j.micpath.2017.10.026 29066377

[B25] Jain S. K. Parsanathan R. Levine S. N. Bocchini J. A. Holick M. F. Vanchiere J. A. (2020). The Potential Link Between Inherited G6PD Deficiency, Oxidative Stress, and Vitamin D Deficiency and the Racial Inequities in Mortality Associated With COVID-19. Free Radic. Biol. Med. 161, 84–91. doi: 10.1016/j.freeradbiomed.2020.10.002 33038530 PMC7539020

[B26] Kesic M. J. Simmons S. O. Bauer R. Jaspers I. (2011). Nrf2 Expression Modifies Influenza A Entry and Replication in Nasal Epithelial Cells. Free Radic. Biol. Med. 51 (2), 444–453. doi: 10.1016/j.freeradbiomed.2011.04.027 21549835 PMC3135631

[B27] Kim S. C. Sprung R. Chen Y. Xu Y. Ball H. Pei J.. (2006). Substrate and Functional Diversity of Lysine Acetylation Revealed by a Proteomics Survey. Mol. Cell. 23 (4), 607–618. doi: 10.1016/j.molcel.2006.06.026 16916647

[B28] Kosmider B. Messier E. M. Janssen W. J. Nahreini P. Wang J. Hartshorn K. L.. (2012). Nrf2 Protects Human Alveolar Epithelial Cells Against Injury Induced by Influenza A Virus. Respir. Res. 13 (1), 43. doi: 10.1186/1465-9921-13-43 22672594 PMC3520784

[B29] Koyuncu E. Budayeva H. G. Miteva Y. V. Ricci D. P. Silhavy T. J. Shenk T.. (2014). Sirtuins Are Evolutionarily Conserved Viral Restriction Factors. mBio 5 (6), e02249–e02214. doi: 10.1128/mBio.02249-14 25516616 PMC4271551

[B30] Lemos V. de Oliveira R. M. Naia L. Szegö É. Ramos E. Pinho S.. (2017). The NAD+-Dependent Deacetylase SIRT2 Attenuates Oxidative Stress and Mitochondrial Dysfunction and Improves Insulin Sensitivity in Hepatocytes. Hum. Mol. Genet. 26 (21), 4105–4117. doi: 10.1093/hmg/ddx298 28973648

[B31] Lu S. C. (2009). Regulation of Glutathione Synthesis. Mol. Aspects. Med. 30 (1-2), 42–59. doi: 10.1016/j.mam.2008.05.005 18601945 PMC2704241

[B32] Lundby A. Lage K. Weinert B. T. Bekker-Jensen D. B. Secher A. Skovgaard T.. (2012). Proteomic Analysis of Lysine Acetylation Sites in Rat Tissues Reveals Organ Specificity and Subcellular Patterns. Cell. Rep. 2 (2), 419–431. doi: 10.1016/j.celrep.2012.07.006 22902405 PMC4103158

[B33] Miri-Aliabad G. Khajeh A. Shahraki T. (2017). Prevalence of G6PD Deficiency in Children With Hepatitis A. Int. J. Hematol. Oncol. Stem Cell. Res. 11 (2), 92–95. 28875002 PMC5575731

[B34] Nencioni L. Iuvara A. Aquilano K. Ciriolo M. R. Cozzolino F. Rotilio G.. (2003). Influenza A Virus Replication Is Dependent on an Antioxidant Pathway That Involves GSH and Bcl-2. FASEB J. 17 (6), 758–760. doi: 10.1096/fj.02-0508fje 12594179

[B35] Outeiro T. F. Kontopoulos E. Altmann S. M. Kufareva I. Strathearn K. E. Amore A. M.. (2007). Sirtuin 2 Inhibitors Rescue Alpha-Synuclein-Mediated Toxicity in Models of Parkinson’s Disease. Science 317 (5837), 516–519. doi: 10.1126/science.1143780 17588900

[B36] Palamara A. T. Nencioni L. Aquilano K. De Chiara G. Hernandez L. Cozzolino F.. (2005). Inhibition of Influenza A Virus Replication by Resveratrol. J. Infect. Dis. 191 (10), 1719–1729. doi: 10.1086/429694 15838800

[B37] Park Y. J. Choe S. S. Sohn J. H. Kim J. B. (2017). The Role of Glucose-6-Phosphate Dehydrogenase in Adipose Tissue Inflammation in Obesity. Adipocyte 6 (2), 147–153. doi: 10.1080/21623945.2017.1288321 28425844 PMC5477698

[B38] Pasquereau S. Nehme Z. Haidar Ahmad S. Daouad F. Van Assche J. Wallet C.. (2021). Resveratrol Inhibits HCoV-229E and SARS-CoV-2 Coronavirus Replication *In Vitro* . Viruses 13 (2), 354. doi: 10.3390/v13020354 33672333 PMC7926471

[B39] Ramezani A. Nahad M. P. Faghihloo E. (2018). The Role of Nrf2 Transcription Factor in Viral Infection. J. Cell. Biochem. 119 (8), 6366–6382. doi: 10.1002/jcb.26897 29737559

[B40] Rojo de la Vega M. Chapman E. Zhang D. D. (2018). N2 and the Hallmarks of Cancer. Cancer Cell. 34 (1), 21–43. doi: 10.1016/j.ccell.2018.03.022 29731393 PMC6039250

[B41] Salvemini F. Franzé A. Iervolino A. Filosa S. Salzano S. Ursini M. V. (1999). Enhanced Glutathione Levels and Oxidoresistance Mediated by Increased Glucose-6-Phosphate Dehydrogenase Expression. J. Biol. Chem. 274 (5), 2750–2757. doi: 10.1074/jbc.274.5.2750 9915806

[B42] Sgarbanti R. Nencioni L. Amatore D. Coluccio P. Fraternale A. Sale P.. (2011). Redox Regulation of the Influenza Hemagglutinin Maturation Process: A New Cell-Mediated Strategy for Anti-Influenza Therapy. Antioxid. Redox Signal. 15 (3), 593–606. doi: 10.1089/ars.2010.3512 21366409

[B43] Shaw P. Chattopadhyay A. (2020). Nrf2-ARE Signaling in Cellular Protection: Mechanism of Action and the Regulatory Mechanisms. J. Cell. Physiol. 235 (4), 3119–3130. doi: 10.1002/jcp.2921 31549397 PMC13482834

[B44] Shytaj I. L. Procopio F. A. Tarek M. Carlon-Andres I. Tang H. Y. Goldman A. R.. (2021). Glycolysis Downregulation Is a Hallmark of HIV-1 Latency and Sensitizes Infected Cells to Oxidative Stress. EMBO Mol. Med. 13 (8), e13901s. doi: 10.15252/emmm.202013901 PMC8350904 34289240

[B45] Simon P. F. McCorrister S. Hu P. Chong P. Silaghi A. Westmacott G.. (2015). Highly Pathogenic H5N1 and Novel H7N9 Influenza A Viruses Induce More Profound Proteomic Host Responses Than Seasonal and Pandemic H1N1 Strains. J. Proteome Res. 14 (11), 4511–4523. doi: 10.1021/acs.jproteome 26381135

[B46] Singh C. K. Chhabra G. Ndiaye M. A. Garcia-Peterson L. M. Mack N. J. Ahmad N. (2018). The Role of Sirtuins in Antioxidant and Redox Signaling. Antioxid. Redox Signal. 28 (8), 643–661. doi: 10.1089/ars.2017.7290 28891317 PMC5824489

[B47] Singh A. Misra V. Thimmulappa R. K. Lee H. Ames S. Hoque M. O.. (2006). Dysfunctional KEAP1-Nrf2 Interaction in Non-Small-Cell Lung Cancer. PLoS Med. 3 (10), e420. doi: 10.1371/journal.pmed.0030420 17020408 PMC1584412

[B48] To E. E. Broughton B. R. Hendricks K. S. Vlahos R. Selemidis S. (2014). Influenza A Virus and TLR7 Activation Potentiate NOX2 Oxidase-Dependent ROS Production in Macrophages. Free Radic. Res. 48 (8), 940–947. doi: 10.3109/10715762.2014.927579 24869957

[B49] van Zwieten R. Verhoeven A. J. Roos D. (2014). Inborn Defects in the Antioxidant Systems of Human Red Blood Cells. Free Radic. Biol. Med. 67, 377–386. doi: 10.1016/j.freeradbiomed.2013.11.022 24316370

[B50] Vick D. J. (2020). Glucose-6-Phosphate Dehydrogenase Deficiency and COVID-19 Infection. Mayo Clin. Proc. 95 (8), 1803–1804. doi: 10.1016/j.mayocp.2020.05.035 32680625 PMC7275177

[B51] Wang M. Hu J. Yan L. Yang Y. He M. Wu M.. (2019). High Glucose-Induced Ubiquitination of G6PD Leads to the Injury of Podocytes. FASEB J. 33 (5), 6296–6310. doi: 10.1096/fj.201801921R 30785802

[B52] Wang Y. P. Zhou L. S. Zhao Y. Z. Wang S. W. Chen L. L. Liu L. X.. (2014). Regulation of G6PD Acetylation by SIRT2 and KAT9 Modulates NADPH Homeostasis and Cell Survival During Oxidative Stress. EMBO J. 33 (12), 1304–1320. doi: 10.1002/embj.201387224 24769394 PMC4194121

[B53] Wu Y. H. Tseng C. P. Cheng M. L. Ho H. Y. Shih S. R. Chiu D. T. (2008). Glucose-6-Phosphate Dehydrogenase Deficiency Enhances Human Coronavirus 229e Infection. J. Infect. Dis. 197 (6), 812–816. doi: 10.1086/528377 18269318 PMC7199897

[B54] Xu Y. Osborne B. W. Stanton R. C. (2005). Diabetes Causes Inhibition of Glucose-6-Phosphate Dehydrogenase *via* Activation of PKA, Which Contributes to Oxidative Stress in Rat Kidney Cortex. Am. J. Physiol. Renal Physiol. 289 (5), F1040–F1047. doi: 10.1152/ajprenal.00076.2005 15956780

[B55] Xu S. N. Wang T. S. Li X. Wang Y. P. (2016). SIRT2 Activates G6PD to Enhance NADPH Production and Promote Leukaemia Cell Proliferation. Sci. Rep. 6, 32734. doi: 10.1038/srep32734 27586085 PMC5009355

[B56] Yang H. C. Wu Y. H. Liu H. Y. Stern A. Chiu D. T. (2016). What has Passed is Prolog: New Cellular and Physiological Roles of G6PD. Free Radic. Res. 50 (10), 1047–1064. doi: 10.1080/10715762.2016.1223296 27684214

[B57] Yang H. C. Wu Y. H. Yen W. C. Liu H. Y. Hwang T. L. Stern A.. (2019). The Redox Role of G6PD in Cell Growth, Cell Death, and Cancer. Cells 8 (9), 1055. doi: 10.3390/cells8091055 PMC6770671 31500396

[B58] Zhao D. Fukuyama S. Sakai-Tagawa Y. Takashita E. Shoemaker J. E. Kawaoka Y. (2015). C646, a Novel P300/CREB-Binding Protein-Specific Inhibitor of Histone Acetyltransferase, Attenuates Influenza A Virus Infection. Antimicrob. Agents Chemother. 60 (3), 1902–1906. doi: 10.1128/AAC.02055-15 26711748 PMC4776003

