# Supplementary figures and images for: Influenza Virus Down-Modulates G6PD Expression and Activity to Induce Oxidative Stress and Promote Its Replication
Source: Front Cell Infect Microbiol. 2022 Jan 6;11:804976. doi: 10.3389/fcimb.2021.804976 (PMC8770543; doi:10.3389/fcimb.2021.804976)

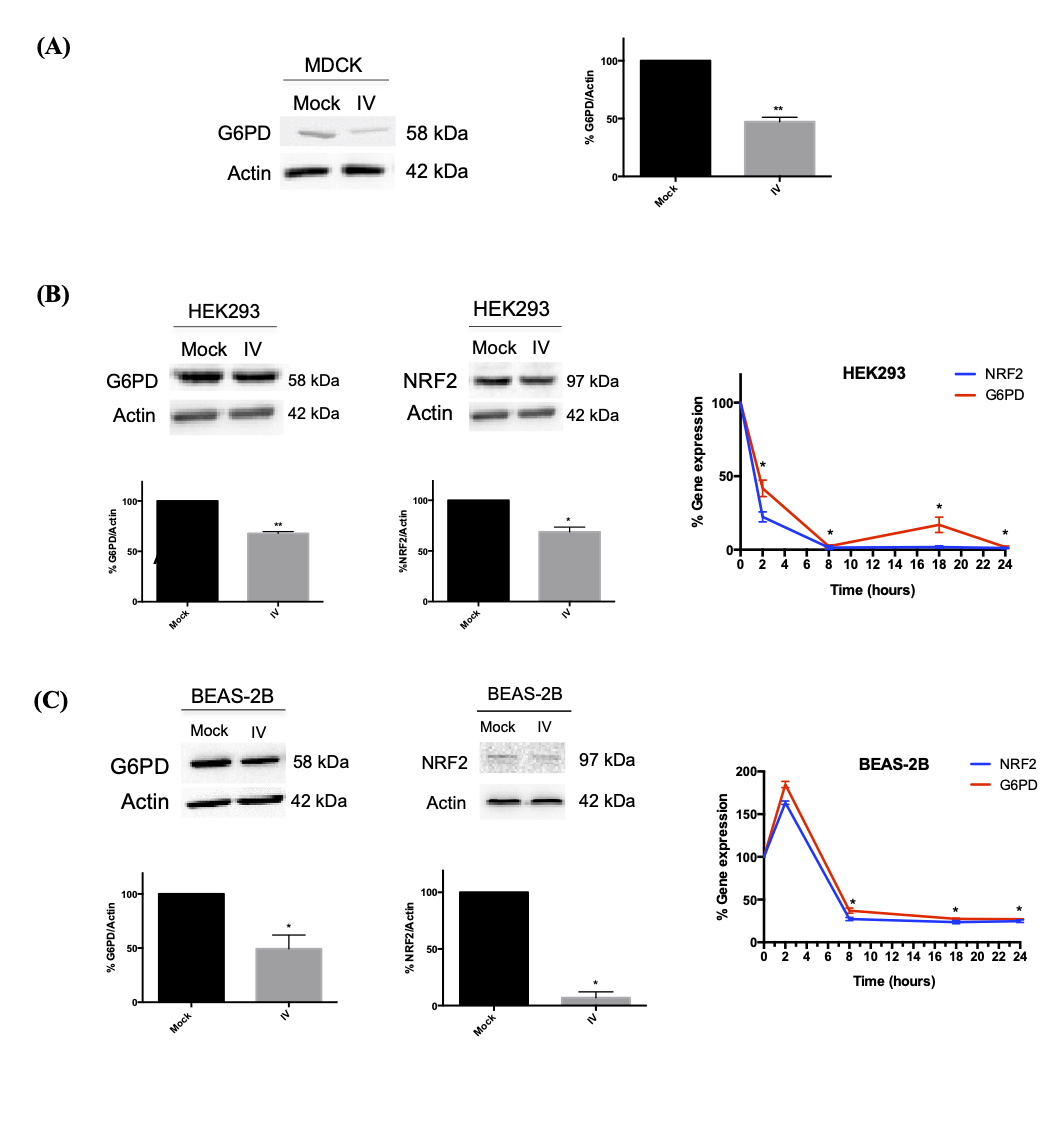

Supplement: Supplementary Figure 1 — Influenza virus infection induces G6PD and NRF2 expression decrease in permissive cell lines. (A) Western blot analysis of G6PD protein expression in MDCK cells infected for 24 h with influenza virus A/PR/8/H1N1 (IV). Actin was used as loading control for the densitometry analysis of two independent experiments. Data are expressed as mean ± S.D (*P < 0.001 vs Mock). (B) Western blot analysis of G6PD and NRF2 protein expression in HEK293 cells infected for 24 h with influenza virus A/PR/8/H1N1 (IV). Actin was used as loading control for the densitometry analysis of two independent experiments. In the right panel, are represented the gene expression analysis of both NRF2 and G6PD during time course experiments (2-24 h). Data are expressed as mean ± S.D. (*P < 0.05 and **P < 0.001 vs Mock). (C) G6PD and NRF2 protein expression in BEAS-2B cell line after 24h of IV PR8 infection. Actin was used as loading control for the densitometry analysis of two independent experiments. In the right panel, are represented the gene expression analysis of both NRF2 and G6PD during time course experiments (2-24 h). Data are expressed as mean ± S.D. (*P < 0.05 vs Mock). [file Image_1.tiff]
